# Mesenteric granulomas independently predict long‐term risk of surgical recurrence in Crohn's disease

**DOI:** 10.1111/codi.14814

**Published:** 2019-08-23

**Authors:** L. W. Unger, S. Argeny, A. Stift, Y. Yang, A. Karall, T. Freilinger, C. Müller, M. Bergmann, J. Stift, S. Riss

**Affiliations:** ^1^ Division of General Surgery Department of Surgery Medical University of Vienna Vienna Austria; ^2^ Clinical Institute of Pathology Medical University of Vienna Vienna Austria

**Keywords:** Inflammatory bowel disease, laparoscopy, intestinal resection

## Abstract

**Aim:**

The risk factors that predict surgical recurrence in Crohn's disease (CD) remain controversial. Postoperative anti‐tumour necrosis factor (anti‐TNF) therapy might lower recurrence rates whilst the presence of mesenteric granulomas has been postulated to increase the risk. We hypothesized that mesenteric granulomas indicate disease severity and might predict the risk of surgical recurrence, irrespective of immunosuppressive therapy.

**Method:**

We performed a retrospective review of all consecutive patients undergoing operations for CD between January 2000 and December 2014 at a single tertiary referral centre and assessed the perioperative factors and histological findings at the time of surgery. Surgical recurrence rates and the immunosuppressive regimen were assessed through retrospective chart review and telephone interviews.

**Results:**

A total of 274 patients were eligible for analysis. Median follow‐up was 8.54 (5.48–14.42) years. A total of 63 patients (23.0%) underwent surgery for recurrent CD after a median of 4.75 (2.10–7.96) years. In final histology, 35 (12.8%) patients had mesenteric granulomas. TNF inhibitors were administered postoperatively in 104 (38.0%) and thiopurines in 137 (50.0%) patients. In univariate analysis, only the presence of mesenteric granulomas [hazard ratio (HR) 1.95; 95% CI 1.05–3.62; *P* = 0.035] significantly increased the risk for recurrent surgery while postoperative anti‐TNF (HR 0.85; 95% CI 0.49–1.50; *P* = 0.581) or thiopurine therapy (HR 1.03; 95% CI 0.61–1.73; *P* = 0.916) did not. In multivariate analysis, only the presence of mesenteric granulomas significantly influenced the risk of surgical recurrence (HR 1.94, 95% CI 1.04–3.60; *P* = 0.037).

**Conclusion:**

Intestinal and mesenteric granulomas should be differentiated in pathology reports, because mesenteric, but not intestinal, granulomas may be associated with an increased risk of surgical recurrence.

**What does this paper add to the literature?**

This paper investigated several established, as well as recently suggested, risk factors for surgical recurrence of Crohn's disease in a large single‐centre long‐term retrospective review. It shows that mesenteric granulomas are an independent risk factor for surgical recurrence while postoperative immunosuppression did not alter the risk of recurrence.

## Introduction

Medical treatment is the main strategy for achieving disease control in Crohn's disease (CD), but the lifetime risk of CD‐related complications that require surgical intervention is still high 1. Owing to the use of thiopurines and biologicals as well as interventional endoscopy, the 5‐year risk of undergoing surgery after diagnosis has significantly declined and is currently around 30% 2. This is partly due to the fact that the gap between diagnosis and initiation of anti‐tumour necrosis factor (anti‐TNF) therapy has decreased 3. Recently, several studies have suggested a favourable outcome with regard to endoscopic and surgical recurrence in patients who receive postoperative anti‐TNF therapy 4, 5, 6, 7. However, these studies were conducted mainly after index ileocolic resection and comprise relatively small sample sizes, warranting further proof‐of‐concept work. Additionally, immunosuppression bears the risk of infection and other adverse events. Therefore, an improved risk assessment for surgical recurrence would potentially allow a more patient‐tailored selection of postoperative immunosuppression.

Several established risk factors such as age, smoking and prior resection contribute to the risk of endoscopic disease recurrence and can only be partially influenced by clinicians 8, 9. Granulomas have been postulated as objective histological indicators of disease severity and thus might indicate an increased risk of postoperative endoscopic and surgical recurrence. However, results remain inconclusive in the available literature 10, 11, 12. These discrepant findings might be due to a lack of differentiation between granuloma site (e.g. intestinal or mesenteric). In a recent work by Li *et al*. 11, postoperative recurrence in 194 patients who underwent index ileocaecal resection for CD‐related complications was analysed according to granuloma site. The authors identified mesenteric lymph node granulomas as an independent prognostic factor for endoscopic as well as surgical recurrence, while intestinal granulomas did not prove valuable as a prognostic factor. These findings are of great interest, as lymphatic vessels are altered in CD and might play a key role in pathogenesis 13. Moreover, mesenteric resection has recently been suggested to potentially reduce surgical recurrence rates 14. Unfortunately, the generalizability of results from index ileocaecal resection is limited. In addition, the utilization of postoperative immunosuppression was relatively low in the published literature. Thus, more data on the prognostic value of objective histological hallmarks in a more general, well‐characterized population are warranted.

Given the available data, we hypothesized that the presence of mesenteric granulomas might serve as an objective marker in a general retrospective cohort of patients and independently predict the risk of surgical recurrence.

## Method

### Setting and participants

We performed a retrospective review of all consecutive patients operated on for CD between 1 January 2000 and 31 December 2014 at the Division of General Surgery, Department of Surgery, Medical University of Vienna and assessed perioperative factors and histological findings at the time of surgery.

After obtaining approval from the Medical University of Vienna's institutional review board, patients were retrospectively identified, and all patients were contacted by post. All eligible subjects were asked to supply written informed consent to participate in the study, as required by the local ethics committee. Telephone interviews and/or questionnaires assessing long‐term surgical recurrence rates and immediate postoperative immunosuppressive regimens were conducted between May 2017 and August 2018.

**Figure 1:**
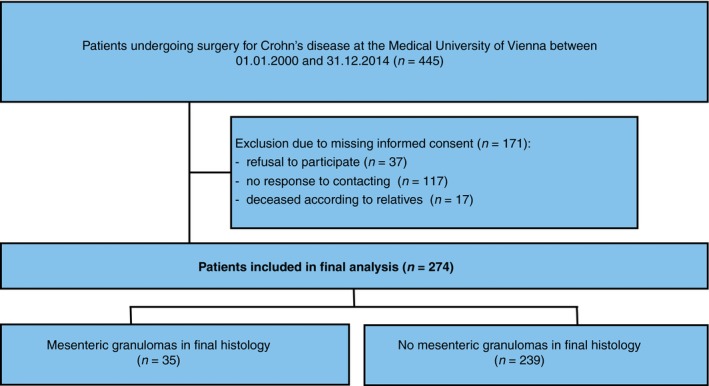
Flow chart depicting patient selection and inclusion as well as exclusion criteria.

Of the 445 patients eligible for inclusion, we had to exclude a total of 171, mainly due to missing informed consent. Detailed information is available in Fig. 1.

### Variables

In order to investigate the influence of several (established) risk factors on long‐term surgical recurrence rates, we assessed sex, age, body mass index (BMI), American Society of Anesthesiologists (ASA) grade, family history of inflammatory bowel disease, number of Crohn's‐related operations and smoking status at the time of surgery between January 2000 and December 2014.

Operative factors such as surgical approach (laparoscopic versus open), urgency of surgery (emergency versus elective), intra‐operative findings (perforation, abscess, stricture) and type of resection were extracted from surgery reports. For analysis of surgical complexity, we defined ‘simple resection’ as only one resection without any additional resection and/or strictureplasty. The presence or absence of mesenteric and/or intestinal granulomas was extracted in a dichotomous fashion (present/absent) from full‐length pathology reports.

### Perioperative outcome and postoperative immunosuppression

Postoperative prophylactic immunosuppressive therapy (thiopurines, steroids, biologicals), postoperative complications (according to the Clavien–Dindo classification 15) and length of hospital stay were extracted from discharge reports and/or outpatient records and data was verified by telephone interviews/questionnaires.

For uni‐ and multivariate analysis, minor complications were defined as Clavien–Dindo grades I and II and major complications were considered as Clavien–Dindo grades III, IV and V.

### Definition of surgical recurrence

Surgical recurrence was defined as the need for any reoperation under general anaesthesia due to recurrence of complicated CD. Balloon dilatations were not considered as surgical recurrence. The date of surgery was assessed by individual chart review and verified via telephone interview/questionnaire to assess surgical recurrence in patients who were followed in other centres.

All patients who were included in the study had been evaluated and discussed in a multidisciplinary team meeting for inflammatory bowel disease prior to surgery.

### Ethical considerations and informed consent

The retrospective study with written informed consent and data verification was conducted in accordance with the Declaration of Helsinki and approved by the local review board (EK numbers 1700/2016 and 1881/2017; access https://ekmeduniwien.at/core/catalog/2016/).

The STrengthening the Reporting of OBservational studies in Epidemiology (STROBE) checklist was used to assure appropriate quality reporting 16.

### Statistical analysis

Continuous variables are reported as median (Q1–Q3) and categorical variables are reported as number (*n*) of patients with the certain characteristic (proportion of patients with the certain characteristic in %). The Mann–Whitney *U*‐test was used as numerical variables were not normally distributed.

Pearson's chi‐square test or Fisher's exact test were used for group comparisons. Characteristics that might affect surgical recurrence‐free survival were analysed using semiparametric Cox proportional hazard models. Univariate Cox regression was carried out to determine factors showing a trend towards improved/impaired surgical recurrence‐free survival. Then all univariate factors with a *P*‐value < 0.1 were included in a separate multivariate model. Patients entered the model on the day of the respective first surgery within the study period and were followed until either the time of CD‐related reoperation (i.e. surgical recurrence; defined as event) or last patient contact (defined as date last seen within the hospital or date of questionnaire return).

**Figure 2:**
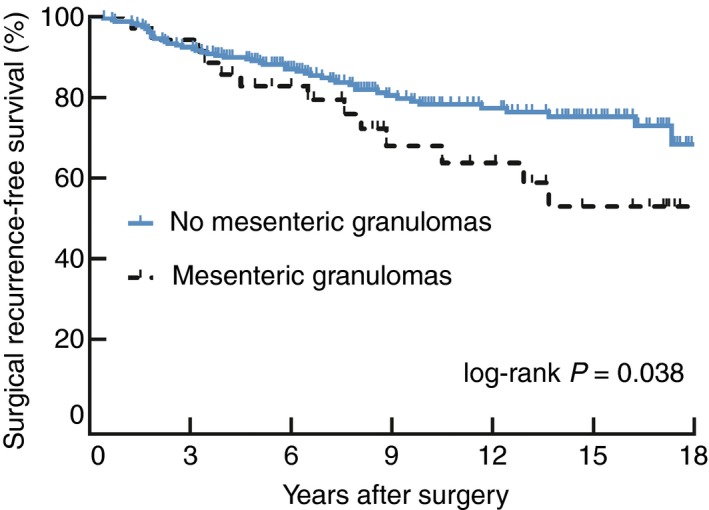
Kaplan–Meier survival curves on surgical recurrence‐free survival. Impact of mesenteric granulomas on risk of surgical recurrence. Patients with mesenteric granulomas in final histology had a significantly higher risk of surgical recurrence during follow‐up (*P* = 0.038).

The log‐rank test was used to identify differences in surgical recurrence‐free survival times between patients with or without mesenteric granulomas (Fig. 2) and patients on different therapies in the respective subgroups (Fig. S1 in the online Supporting Information).

Two‐sided *P*‐values < 0.05 were considered statistically significant.

spss 24.0 (SPSS, Armonk, New York, USA) was used for all statistical analyses. graphpad prism 8 (GraphPad, La Jolla, California, USA) was used for plotting survival curves.

## Results

### Demographic details of patients, perioperative course and postoperative medication

Baseline medical as well as surgical characteristics at the time of surgery are depicted in Table 1. Median follow‐up was 8.54 (5.48–14.42) years. There were slightly more male patients than female ones (52.9% *vs* 47.1%) with a median BMI of 21.50 (19.04–23.96) kg/m². An index operation was performed in 57.3% (157/274) of patients, while 117/274 (42.7%) patients underwent recurrent surgery. In total, 57.3% of patients were operated on via an open approach (open surgery and conversions combined, 157/274). Overall, the majority of operations were elective (248/274, 90.5%).

**Table 1:** Demographic details of Crohn's disease patients, surgical intervention and postoperative medication.

Baseline characteristics	
Female sex	129 (47.1%)
Follow‐up (years)	8.54 (5.48–14.42)
Age (years)	35.20 (26.68–46.03)
Age at CD diagnosis (years)	25.00 (19.00–34.00)
Positive family history	62 (22.6%)
BMI (kg/m²)	21.50 (19.04–23.96)
Smoker at the time of surgery	134 (48.9%)
ASA score (grade)	2 (2–3)
Perioperative characteristics	
First surgery	157 (57.3%)
Second surgery	60 (21.9%)
Third surgery	31 (11.3%)
More than three prior surgeries	26 (9.5%)
Laparoscopic surgery	117 (42.7%)
Open surgery	122 (44.5%)
Conversion during laparoscopic surgery	35 (12.8%)
Intra‐operative perforation	140 (51.1%)
Intra‐operative stenosis	202 (73.8%)
Intra‐operative abscess	68 (24.8%)
Intra‐operative fistula	150 (54.7%)
Duration of surgery (min)	155 (110–195)
Length of hospital stay (days)	9 (8–12)
Elective surgery	248 (90.5%)
Complex resection (more than one resection and/or strictureplasty)	79 (28.8%)
Mesenteric granulomas	35 (12.8%)
Intestinal granulomas	137 (50.0%)
Postoperative characteristics	
Steroids	42 (15.3%)
Azathioprine/6‐mercaptopurine	137 (50.0%)
Anti‐TNF therapy	104 (38.0%)
Recurrent surgery during follow‐up	63 (23.0%)
Time to recurrent surgery (years)	4.75 (2.10–7.96)

Continuous variables are presented as median (Q1–Q3), Frequencies are presented as absolute number (% of total).

ASA, American Society of Anesthesiologists; BMI, body mass index; TNF, tumour necrosis factor.

In addition, the majority of surgical resections were considered simple (195/274, 71.2%), defined as only one resection without any additional resection and/or strictureplasty. In histological work‐up, 50% of the patients (137/274) had intestinal granulomas in the specimen, while only 12.8% (35/274) showed mesenteric granulomas.

A total of 63 patients (23.0%) had to undergo recurrent surgery during follow‐up for CD‐related complications after a median of 4.75 (2.10–7.96) years. Of the 63 patients who were considered as undergoing ‘recurrent surgery during follow‐up’, 3 required subtotal colectomy, 15 underwent subsequent ileocaecal resection, 8 had to undergo anastomotic resection, 18 underwent small bowel resection with or without additional resection (1 had an additional colonic resection due to a stricture and 3 required stoma formation due to perianal disease but without abdominoperineal excision), 13 underwent colonic segmental resection, 3 underwent abdominoperineal excision due to perianal disease and 3 had multiple strictureplasties without resection. The patient characteristics of the respective subgroups are presented in Table S1.

Postoperatively, the most common immunosuppressive regimen consisted of azathioprine/6‐mercaptopurine (137/274, 50%), followed by anti‐TNF‐therapy (104/274, 38.0%). Steroids were only prescribed in rare cases (42/274, 15.3%). Few patients had combinations of immunosuppressants.

### Mesenteric granulomas as the only independent risk factor for recurrent surgery

To assess risk factors for surgical recurrence, univariate and multivariate Cox regression analyses were conducted (Table 2). Published factors that influence postoperative recurrence risk were included in univariate analysis and all variables that showed a trend to influence postoperative surgical recurrence (*P* < 0.1) were included in the multivariate analysis.

**Table 2:** Cox regression analysis on surgical recurrence. A *P*‐value < 0.1 was considered as trend and values are included for multivariate regression analysis.

Patient characteristics	Univariate analysis				Multivariate analysis			
	HR	95% CI		*P*‐value	HR	95% CI		*P*‐value
		Lower	Upper			Lower	Upper	
Sex (male *vs* female)	0.67	0.39	1.12	0.128				
Age at surgery (per patient year)	0.99	0.97	1.01	0.432				
BMI (per kg/m²)	1.01	0.94	1.08	0.735				
ASA grade (per grade)	0.99	0.61	1.62	0.974				
Laparoscopic *vs* open surgery	0.94	0.56	1.59	0.817				
Anti‐TNF postoperatively (yes *vs* no)	0.85	0.49	1.50	0.581				
Steroids postoperatively (yes *vs* no)	0.56	0.24	1.31	0.181				
Azathioprine/6‐mercaptopurine postoperatively (yes *vs* no)	1.03	0.61	1.73	0.916				
Mesenteric granulomas (yes *vs* no)	1.95	1.05	3.62	0.035	1.94	1.04	3.60	0.037
Intestinal granulomas (yes *vs* no)	1.24	0.73	2.09	0.428				
Family history (positive *vs* negative)	1.16	0.64	2.12	0.627				
Emergency *vs* elective surgery	0.82	0.35	1.90	0.638				
Perforating *vs* nonperforating disease	1.05	0.62	1.77	0.858				
Smoking (yes *vs* no)	1.58	0.92	2.705	0.097	1.56	0.91	2.67	0.105
Number of surgeries for CD (per operation)	0.87	0.70	1.09	0.233				
Type of resection (simple resection)								
Large bowel *vs* small bowel	1.13	0.26	4.95	0.871				
Ileocecal *vs* small bowel	0.52	0.12	2.26	0.385				
Complex resection *vs* simple resection*	1.44	0.84	2.46	0.187				
Surgical complications								
Minor *vs* no complications	1.84	0.85	3.96	0.121				
Major *vs* no complications	1.20	0.64	2.26	0.578				

* Complex resection was defined as more than one resection and/or strictureplasty.

ASA, American Society of Anesthesiologists; BMI, body mass index; CD, Crohn's disease; HR, hazard ratio; TNF, tumour necrosis factor.

In univariate analysis, only mesenteric granulomas in final histology [hazard ratio (HR) 1.95; 95% CI 1.05–3.62; *P* = 0.035] and smoking at the time of surgery (HR 1.58; 95% CI 0.92–2.70; *P* = 0.097) showed a trend to affect risk of postoperative recurrence. Postoperative immunosuppressive regimens (anti‐TNF therapy, azathioprine/6‐mercaptopurine or steroids) did not affect recurrence risk, neither did prior operations due to CD, surgical approach or postoperative complications. Therefore, only mesenteric granulomas and smoking at the time of surgery were included in the multivariate analysis. Finally, only the presence of mesenteric granulomas was independently associated with risk of surgical recurrence (HR 1.94; 95% CI 1.04–3.60; *P* = 0.037).

### Differences in patients with or without mesenteric granulomas

To assess differences in surgical recurrence‐free survival in patients with and without mesenteric granulomas, a Kaplan–Meier survival curve was plotted to compare groups (Fig. 2). Recurrence‐free survival after 1, 5 and 10 years was 98.7%, 90.3% and 80.0%, in patients without granulomas and 100%, 82.8% and 68.0%, in patients with mesenteric granulomas in histological work‐up (log‐rank *P* = 0.038). To further identify differences between groups, we outline the baseline characteristics in Table 3. Patients with mesenteric granulomas had a tendency towards younger age compared to patients without granulomas [31.32 (24.10–38.35) years *vs* 36.1 (26.93–46.73) years, respectively; *P* = 0.052] and significantly higher rates of concomitant intestinal granulomas (71.4% *vs* 46.9% in patients with granulomas versus those without granulomas; *P* = 0.010). Moreover, we found a tendency towards more intense immunosuppression postoperatively, reflected by more common use of anti‐TNF therapy (52.9% in patients with mesenteric granulomas versus 36.4% in those without mesenteric granulomas; *P* = 0.089). There were no differences in utilization of azathioprine or steroids. No differences could be found when subgroup analyses of patients on postoperative monotherapy with and without mesenteric granulomas were compared via a log‐rank test (Fig. S1).

**Table 3:** Comparison of perioperative factors at the time of surgery of patients with and without mesenteric granulomas.

Patient characteristics	No mesenteric granulomas (*n* = 239)	Mesenteric granulomas (*n* = 35)	*P*‐value
Female sex	116 (48.5%)	13 (37.1%)	0.277
Age at surgery (years), median (Q1–Q3)	36.1 (26.93–46.73)	31.32 (24.10–38.35)	0.052
BMI (kg/m²), median (Q1–Q3)	21.60 (19.27–23.85)	19.61 (18.07–24.54)	0.216
ASA grade, median (Q1–Q3)	2 (2–3)	2 (2–3)	0.800
Laparoscopic *vs* open surgery	99 (41.4%)	18 (51.4%)	0.278
Steroids postoperatively	36 (15.1%)	6 (17.1%)	0.801
Azathioprine/6‐mercaptopurine postoperatively	119 (49.8%)	18 (51.4%)	1.000
Anti‐TNF postoperatively	86 (36.4%)	16 (52.9%)	0.089
Intestinal granulomas	112 (46.9%)	25 (71.4%)	0.010
Positive family history	53 (22.4%)	9 (25.7%)	0.668
Emergency *vs* elective surgery	24 (10.0%)	2 (5.7%)	0.549
Perforating *vs* non‐perforating disease	125 (52.3%)	15 (42.9%)	0.366
Active smoker at surgery	116 (49.6%)	18 (51.4%)	0.858
Type of resection*			
Small bowel resection	48 (20.1%)	6 (17.1%)	0.822
Ileocecal resection	156 (65.3%)	24 (68.6%)	0.849
Large bowel resection	83 (34.7%)	10 (28.6%)	0.849
Strictureplasty	27 (11.3%)	6 (17.1%)	0.401
Postoperative complications			
Clavien–Dindo I	15 (6.3%)	1 (2.9%)	0.985
Clavien–Dindo II	45 (18.8%)	7 (20.0%)	
Clavien–Dindo III	16 (6.7%)	2 (5.7%)	
Clavien–Dindo IV	3 (1.3%)	0 (0.0%)	
Clavien–Dindo V	2 (0.8%)	0 (0.0%)	

* Seventy‐nine patients had more than one resection and/or strictureplasty during the same surgery.

ASA, American Society of Anesthesiologists; BMI, body mass index; CD, Crohn's disease; TNF, tumour necrosis factor.

## Discussion and conclusion

In this retrospective single‐centre cohort study we have investigated several established as well as recently suggested risk factors for surgical recurrence of CD. In the general cohort of patients investigated, only granulomas found in the mesentery of resected specimens predicted the risk of surgical recurrence while other patient‐specific and treatment‐specific factors did not. As utilization of this recently suggested risk factor could help to identify high‐risk patients, these findings must be put into the context of the available literature.

Due to the specialized care required for CD patients, surgery is commonly deferred. Abdominal operations remain the ‘last resort’ for more complex disease courses only after several conservative approaches have failed 17. Despite common utilization of minimally invasive approaches 18, surgery‐related morbidity remains. In addition, while many studies have investigated immunosuppressants after index ileocaecal resection, limited evidence is available on the optimal immunosuppressive regimen in CD patients with refractory disease who require recurrent surgery. Moreover, trials that evaluate anti‐TNF therapy tend to be focused on endoscopic rather than surgical recurrence 19.

This gap in available data highlights the need for evidence‐based treatment approaches since (cumulative) digestive damage might be underestimated by established scores such as the CD activity index 20. Thus, due to the variance in disease course, objective histological hallmarks for grading disease severity would be favourable for decision‐making 8. A recent study conducted by Randolph *et al*. 13 has revived interest in the alteration of lymphatic vessels in CD and shown that B‐cell‐rich aggregates resembling tertiary lymphoid organs impinge on lymphatic collecting vessels adjacent to mesenteric lymph nodes. These alterations might be causative or a result of secondary changes due to chronic inflammation. With respect to this, more extensive resection of the mesentery has been suggested to decrease rates of surgical recurrence after ileocolic resection, although a prospective evaluation of these results is lacking to date 14. Taken together, granulomas in the mesentery might be caused by excessive inflammation and their presence or absence could allow ‘grading’ of disease severity. In contrast, granulomas in the bowel wall seem to be less specific for a complicated disease course/disease duration. This is in line with a meta‐analysis that found higher reoperation rates and faster recurrence in granulomatous CD 12. Using a more differentiated approach, Li *et al*. 11 recently showed that mesenteric granulomas had a prognostic impact on endoscopic and surgical recurrence after index ileocaecal resection while intestinal granulomas did not. However, the postoperative utilization of immunosuppression was relatively low in their study and the impact on recurrence rate is not stated. In our study we have independently validated the findings of Li *et al*. in a larger and more generalized cohort of CD patients. However, the higher utilization rate of anti‐TNF therapy in did not reduce the risk of recurrence in our retrospective cohort. These findings are in line with a nationwide cohort study from Denmark that evaluated a total of 48 967 individuals and did not find a ‘convincing surgery‐sparing effect’ of higher rates of use of immunosuppression 21. In contrast, several prospective studies in small cohorts of patients undergoing index ileocaecal resection have shown benefits for early initiation of anti‐TNF therapy with regard to endoscopic and surgical recurrence rates 5, 6, 7, 22. These discrepant findings in the published literature can probably be attributed to the different inclusion criteria, and collectively indicate that future studies should focus on improving reoperation rates in high‐risk patients. In addition, these discrepancies between ‘real‐world’ studies and controlled trials emphasize the need for studies in patients suffering from more complicated diseases (e.g. a second or third surgical intervention).

Of note, our study has some limitations that need to be addressed. Firstly, we had to exclude a significant number of patients from the analysis (171/445, 38.4%) due to missing informed consent to participate in the study, which was required by the institutional review board. Therefore, we cannot rule out a selection bias. Secondly, despite prospective telephone interviews and confirmation of our compiled data, the study remains a retrospective one with all the associated limitations. In particular, switching of immunosuppressants within the follow‐up period and patient noncompliance cannot completely be ruled out. Due to the impossibility of reliably assessing endoscopic recurrence/interventions in this setting, only surgery under general anaesthesia was considered as ‘surgical recurrence’ while balloon dilatation, for example, was not. Thirdly, although CD histology is evaluated by specialized colorectal pathologists in our centre and granulomas are usually reported in the final reports, the mesentery is not extensively resected in our centre and some granulomas that are located more distant to the bowel wall could potentially be missed. Finally, our tertiary referral centre has a high rate of complex disease courses and patients with medically refractory disease, potentially limiting the applicability to other centres.

In conclusion, mesenteric but not intestinal granulomas reflect disease severity and should trigger closer monitoring of affected patients. While immunosuppression did not alter the risk of surgical recurrence in the presented population, future studies must focus on patient‐tailored approaches to further reduce recurrent surgery rates.

## Conflicts of interest

None of the authors has to declare any conflicts of interest regarding the study.

## Author contribution

LWU and SA designed the study, acquired, analysed and interpreted the data and drafted the manuscript. AS, M, JS and SR contributed to study conception and design. YY, AK and TF performed data acquisition. CM performed data analysis and interpretation. All authors critically revised the manuscript for intellectual content, approved the final version of the manuscript and agree to be accountable for all aspects of the work to ensure that questions regarding accuracy and integrity are investigated and resolved.

## Supporting information


**Table S1**. Patient characteristics according to surgical recurrence status.
**Figure S1.** Subgroup analysis of patients receiving immunosuppressive monotherapy or no therapy after surgical resection.

## Data Availability

All relevant data are presented within the manuscript. The datasets generated and/or analysed in the current study are not publicly available due to patient privacy but are available from the corresponding author on reasonable request.
