# Supplementary material for: Mesenteric granulomas independently predict long‐term risk of surgical recurrence in Crohn's disease
Source: Colorectal Dis. 2019 Aug 23;22(2):170–7. doi: 10.1111/codi.14814 (PMC7028106; doi:10.1111/codi.14814)
Supplement: Supplementary file 1 — Table S1. Patient characteristics according to surgical recurrence status. Figure S1. Subgroup analysis of patients receiving immunosuppressive monotherapy or no therapy after surgical resection. [file CODI-22-170-s001.docx]

Supplementary data

**Mesenteric granulomas independently predict long-term surgical recurrence risk in Crohn’s disease**

L.W. Unger, M.D., PhD, S. Argeny, M.D., A. Stift, M.D., Y. Yang, cand. med., A. Karall, cand.med., T. Freilinger, cand.med., C. Müller, M.D., M. Bergmann, M.D., J. Stift, M.D., and S. Riss, M.D., FRCS

| **Patient characteristics** | **No surgical recurrence**  **(n=211)** | **Surgical recurrence (n=63)** |
| --- | --- | --- |
| Female sex | 93 (44.1%) | 36 (57.1%) |
| Age at surgery [Years]; Median (Q1-Q3) | 35.40 (26.7-46.7) | 33.94 (27.25-40.29) |
| BMI [kg/m²]; Median (Q1-Q3) | 21.41 (19.03-23.85) | 21.72 (19.04-24.60) |
| ASA Grade; Median (Q1-Q3) | 2 (2-3) | 2 (2-3) |
| Laparoscopic surgery | 90 (42.7%) | 27 (42.9%) |
| Steroids postoperatively | 34 (16.1%) | 8 (12.7%) |
| Thiopurine postoperatively | 105 (49.8%) | 32 (50.8%) |
| Anti-TNF postoperatively | 85 (40.3%) | 19 (30.2%) |
| Mesenteric granulomas | 22 (10.4%) | 13 (20.6%) |
| Intestinal granulomas | 103 (48.8%) | 34 (54.0%) |
| Positive family history | 48 (22.7%) | 14 (22.2%) |
| Elective surgery | 194 (91.9%) | 54 (85.7%) |
| Perforating disease | 106 (50.2%) | 34 (54.0%) |
| Active Smoker at Surgery | 96 (45.5%) | 38 (60.3%) |
| ***Type of resection**** |  |  |
| Small bowel resection | 39 (18.5%) | 15 (23.8%) |
| Ileocecal resection | 144 (68.2%) | 36 (57.1%) |
| Large bowel resection | 69 (28.4%) | 24 (38.1%) |
| Strictureplasty | 24 (11.4%) | 9 (14.3%) |

**Supplementary table 1: Patient characteristics according to surgical recurrence status.**

*******79 patients had >1 resections and/or strictureplasties during the same surgery.

**Supplementary Figure 1. Subgroup analysis of patients receiving immunosuppressive monotherapy or no therapy after surgical resection. (**A) Overall cohort of patients on no therapy or monotherapy. (B) Subgroup analysis of patients on no therapy or monotherapy without mesenteric granulomas in final histology. (C) Subgroup analysis of patients on no therapy or monotherapy with mesenteric granulomas in final histology.
